# Optimization, comparison and mechanism of ultrasound-assisted cellulase hydrolysis and ethanol extraction of quercetin, luteolin and apigenin from male inflorescences of *Populus alba* × *berolinensis*

**DOI:** 10.1016/j.ultsonch.2025.107645

**Published:** 2025-10-22

**Authors:** Ru Zhao, Xiaoli Li, Xiuqi Wu, Yulong Wu, Ning Tang, Chen Xu, Tingli Liu, Ailing Ben

**Affiliations:** a Nanjing Engineering Research Center for Peanut Genetic Engineering Breeding and Industrialization, School of Food Science, Nanjing Xiaozhuang University, Nanjing 211171, China; b Department of Biology, University of North Carolina at Chapel Hill, Chapel Hill, NC 27599-3280, USA

**Keywords:** Male inflorescences of *Populus alba* × *berolinensis*, Ultrasound-assisted cellulase hydrolysis, Quercetin, Luteolin, Apigenin

## Abstract

In this study, we aimed to determine the optimal process parameters for extracting quercetin, luteolin and apigenin from the male inflorescences of *Populus alba* × *berolinensis* via the ultrasound-assisted cellulase hydrolysis and ethanol extraction (UACHEE) method and investigated the mechanism of cellulase action during the extraction process. The Box–Behnken design (BBD) was subsequently used to optimize the operating parameters. The optimal extraction parameters of ultrasound-assisted cellulase hydrolysis were as follows: ethanol volume fraction of 70 %, 40 mg/g of dose of cellulase, 50 °C of incubation temperature, 148 min of incubation time, pH of 5, mL/g of liquid‒solid ratio, duty cycle of 16.67 %, 200 W of ultrasound irradiation power in the incubation process, 200 W of ultrasound irradiation power in the extraction process, and 15 min of ultrasound irradiation time in the extraction process. Compared with other extraction methods, UACHEE presented an advantage in maintaining higher yields of quercetin, luteolin and apigenin (428.68, 549.65, 1136.20 μg/g). In addition, we used different characterization methods to clarify the extraction mechanism of this method to better improve the extraction yield and efficiency of the target components. This study will lay a theoretical foundation for the potential practical application of the male inflorescences of *P. alba × berolinensis* extracts in fields such as medicine, functional foods, cosmetics, environment and agriculture.

## Highlights

• Ultrasound-assisted cellulase hydrolysis and ethanol extraction (UACHEE) was used.
• Male inflorescence of *Populus alba* × *berolinensis* was applied to extracting materials.
• Quercetin, luteolin and apigenin were simultaneously obtained.
• Compared with other methods, UACHEE has higher extracting yield and efficiency.
• The possible mechanism of UACHEE extraction was explored and discussed.

## 1 Introduction

*Populus alba × berolinensis* is an excellent artificial hybrid of tree species with *Populus alba* as the female parent and *Populus berolinensis* as the male parent, which was cultivated by the Shelter-Forest Research Institute of Heilongjiang Province in the 1980s [1]. *P. alba* × *berolinensis* is a male clone, and the length of its male inflorescence is approximately 5 cm. In addition, *P. alba* × *berolinensis* has several characteristics, such as a tree-like appearance, fast growth, disease resistance, drought resistance, cold tolerance, salt tolerance and beautifying environment [2]. It is not only an excellent tree species for the construction of fast-growing and high-yield forests but also a preferred species for protecting forests and making green areas for cities and countryside areas in Northeast, Northwest and North China [3]. This process can prevent dust from spreading and absorbing harmful gases to reduce air pollution through microclimate regulation, which contributes to ecological protection and construction [4,5]. At present, the use of poplars generally focuses only on wood, but many male inflorescences of *P. alba* × *berolinensis* fall in spring, which affects the beauty of cities and prevents them from being fully utilized [6].

Research has reported that the male inflorescences of *Flos populi* contain glycosides, cardiotonics, flavanoids and phenols [7,8]. There are further studies on the adsorption and desorption characteristics of macroporous resins for quercetin, luteolin and apigenin from *Flos populi* [9]. We also extracted some flavonoid ingredients, such as quercetin, luteolin, and apigenin from the male inflorescence of *P. alba* × *berolinensis*. The structures of the three flavonoids are presented in Fig. S1. Quercetin has been reported to have various therapeutic applications, such as anticancer, anti-inflammatory, antiobesity, arthritis, allergy and asthma treatments [10,11]. Further investigations indicated that quercetin also has other applications for inducing visual monitoring of fish spoilage [12], regulation of glucolipid metabolism disorders [13], cell protection via erythropoietin [14], cutaneous wound healing [10], and anti-inflammatory and analgesic drugs [15]. Recently, luteolin, a Chinese herb found in many fruits and green plants [16], has caused widespread concern due to its pharmacological effects, such as anticancer potential [17], hepatoprotective [18], reducing inflammation [19], neuroprotection [20], antioxidant and antitumor effects [21]. Some studies have indicated that apigenin has various pharmacological activities, including anticancer [22], anti-inflammatory and antioxidant activities [11]. Quercetin, luteolin and apigenin, as natural ingredients, have become popular research topics.

At present, some conventional methods for extracting these active ingredients include conventional solvent extraction, dipping, heat reflux extraction, boiling, and percolation [23]. In addition, several new methods, such as supercritical fluid extraction [24], ultrasonic-assisted extraction [25], microwave-assisted extraction [26], enzymatic hydrolysis [27], accelerated solvent extraction [28], have also been applied. However, supercritical fluid extraction is suitable only for low-level molecular substances [29]. The enzymatic hydrolysis process takes a long time (1–24 h), and cellulase is a macromolecular substance with poor permeability, which results in an incomplete hydrolysis process. Previous studies have demonstrated that ultrasound-assisted extraction, as an emerging and effective extraction technique, is a superior technology for extracting various active components [30,31] because it is easy to perform, takes less time (20–40 min), consumes less solvent, saves energy, is more efficient and has environmentally friendly advantages.

In addition, the plant cell wall is composed of macromolecular substances such as cellulose, hemicellulose and pectin, which obstruct the target components of small-molecule extracts and dissolve. Therefore, enzymatic hydrolysis combined with ultrasonic extraction may be an effective approach for extracting these target components. The glycosidic bonds of polysaccharide macromolecules and flavonoid glycosides are hydrolyzed and cleaved by celulase, which accelerates the extraction and dissolution of the target components of small molecules. Furthermore, enzymatic methods can disrupt the cell wall with several advantages, such as highly sensitive reactions at low temperature and atmospheric pressure, mild treatment conditions, reduced energy consumption, and the production of many high-value products [32].

On the basis of previous studies, the combination of ultrasonication and enzymatic methods mostly involves the addition of organic solvents such as ethanol, the filtration of the supernatant after the enzymatic hydrolysis process, and the direct measurement of the target components of the supernatant [7]. There is no doubt that water, as the most ideal “green solvent”, has been applied to extract polyphenols, flavonoids or other bioactive compounds, which diminishes environmental pollution and may avoid safety problems [33]. Unfortunately, bioactive compounds have a limited solubility in water because of their different properties and thus hinder the application of aqueous solvents to green separation [34]. Because flavonoid aglycones have poor solubility in water, most flavonoids are not fully extracted from material residues. More effectively, some micromolecular components, such as flavonoid aglycones, are extracted by organic solvents such as ethanol. However, the enzyme-encoding generally performs a catalytic role in the aqueous phase, and the enzyme is deactivated with the addition of ethanol, which indicates that it is necessary to extract it again from material residues via ethanol. Therefore, ultrasound-assisted cellulase hydrolysis and ethanol extraction are used to obtain quercetin, luteolin and apigenin. The integration of ultrasound and enzymatic hydrolysis represents a sustainable process-intensification strategy that reduces energy consumption and solvent use.

In the present study, we aimed to develop an efficient ultrasound-assisted cellulase hydrolysis and ethanol extraction (UACHEE) approach for preparing quercetin, luteolin, and apigenin from the male inflorescence of *Populus alba* × *berolinensis*. The operational conditions affecting the yields (ethanol volume fraction, dose of cellulase, incubation temperature and time, pH, liquid‒solid ratio, duty cycle, ultrasonic irradiation power during the incubation process, ultrasonic irradiation power and time during the extraction process) were systematically explored. These parameters were further optimized via the Box–Behnken design (BBD). Scanning electron microscopy (SEM), X-ray diffraction (XRD), and Fourier transform infrared (FTIR) spectroscopy were used to explore the working mechanism of ultrasound-assisted cellulase hydrolysis.

## 2 Materials and methods

### 2.1 Raw materials and reagents

Male inflorescences of *P. alba × berolinensis* were obtained in April 2023 from Harbin, Heilongjiang Province, Northeast China, and were authenticated by Prof. Ailing Ben from Nanjing Xiaozhuang University, China. The plant specimen was stored in the herbarium of Key Construction Laboratories of Pro-vincial Universities for the Resource Utilization of Food and Drug Substances (specimen number: PB20240401). The appearance of male inflorescences of *P. alba × berolinensis* is shown in Fig. S2. Before the experiments were started, the raw materials were crushed with a disintegrator and then sieved with a 60 mesh sieve. The crushed sample was preserved in an airtight container with a cool and dry environment for all the experiments. The moisture content of the male inflorescences of *P. alba × berolinensis* was measured as 6.59 %.

The reference substances quercetin, luteolin, apigenin (purity > 98 %) and cellulase (50 U/mg) were purchased from Shanghai Yuanye Biotechnology Co., Ltd. (Shanghai, China). Methanol, phosphoric acid and ethanol were acquired from Sinopharm Group Chemical Reagent Co., Ltd. (Shanghai, China). The mobile phase was prepared with methanol and 0.1 % phosphoric acid. Before quercetin, luteolin and apigenin were eluted by qualitative and quantitative high-performance liquid chromatography (HPLC), they were filtered through a 0.45 μm nylon 6 membrane. Potassium bromide for FTIR was of spectral purity and purchased from Tianjin Komiou Chemical Reagent Co., Ltd. (Tianjin, China).

### 2.2 Ultraviolet full-wavelength scanning of reference stock solutions

**Fig. 1:**
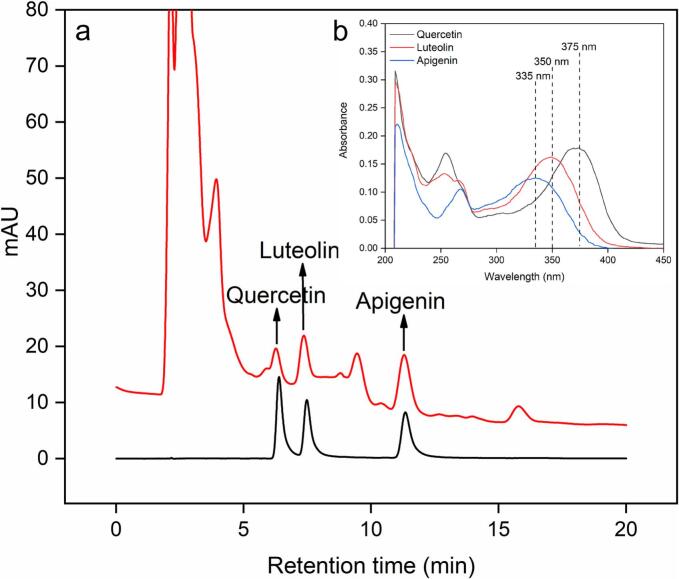
HPLC results for quercetin, luteolin and apigenin in the extraction solution, the red line represents the sample extraction solution, and the black line represents the reference substance solution (a). Insert: UV full wavelength scanning of quercetin, luteolin and apigenin (b).

Standard stock solutions of quercetin, luteolin and apigenin were prepared with methanol, respectively, and then stored in a refrigerator at 4 °C for HPLC detection. The three standard stock solutions were subsequently diluted 25 times for full-wavelength scanning via a UV-5500PC UV‒visible spectrophotometer (Yuanxi, Shanghai, China). The main technical indicators used were as follows: scanning range of 200–900 nm, bandwidth of 1 nm, wavelength accuracy of ±0.1 nm, wavelength repeatability < 0.05 nm, photometer accuracy of ±0.003 A, photometer repeatability < 0.001 A, baseline drift < 0.0001 A/h, and noise < 0.00008 A. The full-wavelength scanning results for quercetin, luteolin and apigenin are shown in Fig. 1b. The optimal absorption wavelengths of quercetin, luteolin and apigenin were respectively 375, 350, and 335 nm, which were the quantitative wavelength for three target components detected by HPLC.

### 2.3 Ultrasound-assisted cellulase hydrolysis and ethanol extraction procedure for quercetin, luteolin and apigenin

The entire experimental operation was completed via a KQ-250DB ultrasonic bath (Kunshan Ultrasonic Instrument Co., Ltd, Kunshan, China) in combination with a constant-temperature oscillating water bath. The KQ-250DB ultrasonication equipped with frequency of 40 kHz and power (100, 150, 200, and 250 W). The ultrasonic capacity was 10 L and the temperature of the ultrasonic bath was maintained by continuously pumping in and out thermostatic water. First, 0.5 g of raw material powder was placed into a 25 mL round bottom plastic centrifuge tube, and cellulase solution was added (the pH of the cellulase solution was adjusted to 5.0 with NaOH solution before enzymatic hydrolysis). The above mixture was incubated for 120 min at 50 °C, and the duty cycle was adjusted during the incubation process. When the incubation process was finished, the filtrate was quickly collected and filtered, and the filtrate volume was measured. The filter residue was supplemented with absolute ethanol such that the ethanol reached a certain concentration. Then, the ultrasonic extraction process was carried out for a certain time. The supernatant was mixed thoroughly, filtered through a 0.45 µm organic filter and then stored at 4 °C until it was used to determine the flavonoid content (quercetin, luteolin and apigenin) via HPLC and repeat 3 times. All experiments were performed in triplicate and data expressed as mean ± SD.

### 2.4 HPLC apparatus and quantitative conditions

The Agilent 1260 Chromatography System (Agilent, Santa Clara, California, USA) consists of an online degassing unit, a quaternary pump, an autosampler, a thermostatic chamber and a diode-array detector (DAD) for the determination of quercetin, luteolin, and apigenin. Chromatographic separation was performed on a Hypersil BDS-C18 reversed-phase column (AkzoNobel, Bohus, Sweden). The detailed determination conditions followed Chen’s method with slight modifications [35], which are as follows. Quercetin, luteolin, apigenin were detected at a wavelength of 375 nm, 350 nm, and 335 nm, respectively. Methanol-water-phosphoric acid (45:54.5:0.5, v/v/v) was used as the mobile phase at a flow rate of 1.0 mL/min. 20 μL of sample was injected for each separation and the running time was 20 min. Under the detecting conditions mentioned above, quercetin, luteolin and apigenin were baseline separated, with the retention time of 6.36 min for quercetin, 7.44 min for luteolin and 11.31 min for apigenin. The identification of quercetin, luteolin, and apigenin was established by matching their chromatographic retention times with those of authentic standards, which are presented in Fig. 1a.

The calibration curves of quercetin, luteolin and apigenin, namely, the relationships between their peak areas (*Y*) and concentrations (*X*), were generated via the standard addition method. The regression linear equation of the calibration curve for quercetin (*Y_1_*), luteolin (*Y_2_*) and apigenin (*Y_3_*) was *Y_1_* = 9.9893 *X*  + 15.336 (*R^2^* = 0.9992, n = 5), *Y_2_* = 9.4694 *X*  + 9.8623 (*R^2^* = 0.9961, n = 5) and *Y_3_* = 9.9806 *X* - 4.6006 (*R^2^* = 1, n = 5), which indicated good linearity of the calibration curves for quercetin, luteolin and apigenin over the range of 0.01–2.00 mg/mL.

### 2.5 Box–Behnken design of the RSM

To further refine the extraction process and predict the optimal conditions for maximizing yield, a Box–Behnken design (BBD) was employed. The experiments were designed and analyzed via Design Expert software (version 8.0). Building upon preliminary single-factor experiments, this study utilized a three-factor response surface methodology (RSM) to model the relationships between process variables and response outputs, thereby optimizing the extraction parameters. To elucidate the interplay between key process variables and response outputs and ultimately identify optimal extraction parameters, this study implemented a three-factor RSM. This approach was informed by prior single-factor experiments. The doses of cellulase (*X_1_*: 10, 25, 40 mg/g), incubation temperature (*X_2_*: 40, 50, 60 °C) and incubation time (*X_3_*: 90, 120, 150 min) were selected to BBD to investigate their interaction influence on the yields of quercetin, luteolin and apigenin. The yields of quercetin, luteolin and apigenin were variable. The experimental design comprised 17 total runs, including 5 center-point replicates and 12 factorial points, to enable pure error estimation. All trials were randomized to reduce systematic error impacts, with triplicate measurements of HPLC for each condition.

### 2.6 Comparison with other methods including ultrasonic-assisted extraction and heat reflux extraction

The ultrasound-assisted cellulase hydrolysis and ethanol extraction (UACHEE) approach was used to extract target components from *P. alba × berolinensis*, which was compared with other methods. The ultrasonic-assisted extraction (UAE) method included cellulase pretreatment with pure water or 60 % ethanol, and the other steps were the same as those described in Section 2.3. The conventional methods of heat reflux extraction (HRE), including cellulase pretreatment with pure water or 60 % ethanol, were used for comparison, and the specific steps were as follows. A total of 10 g of material was taken into a round-bottom flask, and then 200 mL of pure water or 60 % ethanol was added. The round-bottom flask was connected to a reflux condenser and then placed into the HDM-500D digital display heating and temperature control heating sleeve (Jiangsu Ronaldo Instrument Manufacturing Co., Ltd., Changzhou, China) and extracted for 2 h. After the extraction process, the super extract was filtered through a 0.45 μm membrane, and the contents of quercetin, luteolin and apigenin were determined by HPLC.

### 2.7 Method validation

The performance of the proposed UACHEE method was assessed using HPLC under optimized analytical conditions. Key validation parameters, such as linearity, detection limit (LOD), quantification limit (LOQ), reproducibility, stability, recovery, and precision. The LOD for shikimic acid was derived from the standard deviation of the regression line’s Y-intercept (α) and the slope (S), calculated as LOD = 3.3α/S. Similarly, the LOQ was determined using the formula LOQ = 10α/S, ensuring accurate sensitivity measurements under the specified chromatographic conditions.

### 2.8 Scanning electron microscopy (SEM) analysis

The morphological alterations of male inflorescences of *Populus alba × berolinensis* samples before and after treatment (ultrasonic extraction or enzymatic hydrolysis) were scanned via a JSM-7500F scanning electron microscope (Japanese Electronics Corporation, Tokyo, Japan). The main technical indicators are as follows: Gentle Beam and R-filter functions; platinum-gold target spray gold; magnification: 25–1000000; and acceleration voltage: 0.1–30 kV. Samples were fixed on a specimen holder with conductive tape and sputtered with a thin layer of gold, and examined at an accelerating voltage of 20 kV under high vacuum condition (500 × magnification).

### 2.9 Fourier transform infrared spectroscopy (FTIR) analysis

A Fourier transform infrared spectrometer (ThermoFisher, Massachusetts, USA) was used to obtain the spectra of the samples in the 4000–400 cm^−1^ scanning wavelength range. Two hundred milligrams of potassium bromide and 2 mg of sample powder were mixed evenly and ground adequately in an agate mortar. The prepared powder was put into the mold and pressed for 8 min.

### 2.10 X-ray diffraction (XRD) analysis

A D/max-2200VPC X-ray diffraction analyzer (Nippon Science Corporation, Tokyo, Japan) was used to measure the crystallinity of the samples. The main technical indicators used were a scanning range (2*θ)* of 5–60°, a maximum power of 2 kW, a rated voltage of 20–60 kV, a rated current of 12–50 mA, and a scanning speed of 5°/min.

## 3 Results and discussion

### 3.1 Effect of the ethanol volume fraction

**Fig. 2:**
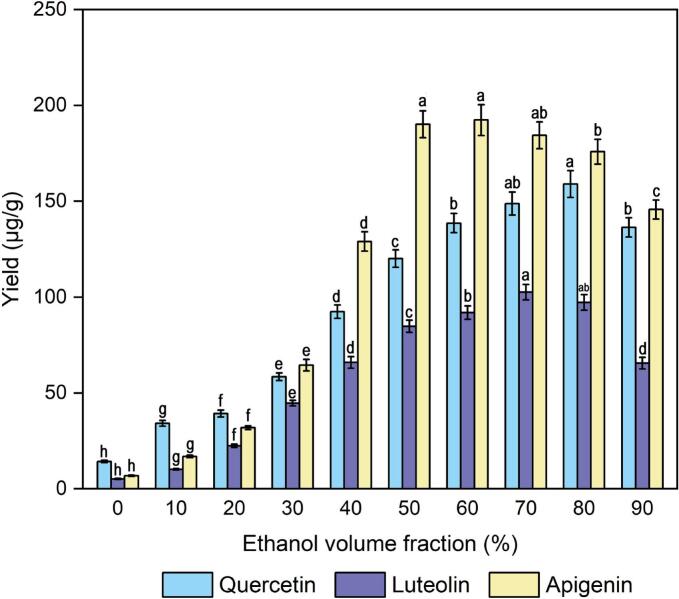
Effect of the ethanol volume fraction on the extraction yields of quercetin, luteolin and apigenin.

The ethanol volume fraction is one of the major factors affecting the yields of quercetin, luteolin and apigenin. The ethanol volume fraction was varied from 0 % to 90 %, and the other experimental conditions were held constant. As shown in Fig. 2, the yields of quercetin, luteolin and apigenin were respectively 14.23, 5.16, and 6.83 μg/g when the volume fraction was 0 % and a similar phenomenon was reported in Echeverry’s study [36]. When the volume fraction of ethanol was over 0 % and increased to 70 %, the yields of quercetin, luteolin and apigenin gradually increased. In contrast, the yields had the decreased trend with increasing ethanol volume fraction 70 %–90 %, which may be attributed that too high of an ethanol volume fraction has the possibility for dehydration of internal plant cells and protein denaturation of cell walls, and thus prevents the dissolution of target components [37]. Ethanol reduces the dielectric constant of the extraction solvent and further promotes the solubility and diffusion of components [37]. The same trends have also been reported in other studies on different ethanol volume fractions for the extraction of flavonoids [35]. This phenomenon suggested that the diffusion and dissolution of three compounds from male inflorescences of *Populus alba × berolinensis* with 70 % ethanol volume fraction were greater than those of the other fractions. Ethanol helps reduce the dielectric constant of the extraction solvent and further promotes the solubility and diffusion of components [38,39]. Therefore, the ethanol concentration was a significant limiting factor affecting the yields of quercetin, luteolin and apigenin. Finally, 70 % of the volume fraction, as the best condition, was selected for follow-up experiments.

### 3.2 Effect of the dose of cellulase

**Fig. 3:**
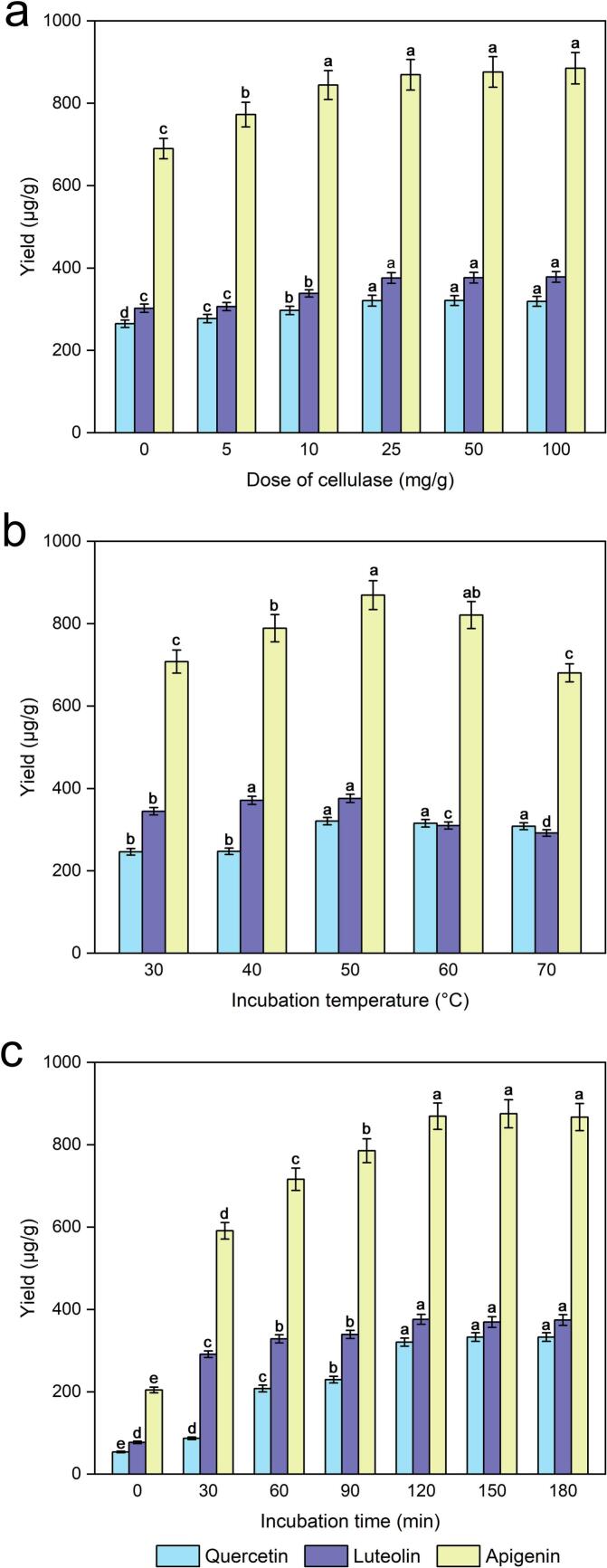
Effect of the dose of cellulase (a), incubation temperature (b), and incubation time (c) on the extraction yields of quercetin, luteolin and apigenin.

To investigate the influence of dose of cellulase on the yields of quercetin, luteolin and apigenin, the set of experiments were performed with different doses of cellulase (0, 5, 10, 25, 50, or 100 mg/g materials). As shown in Fig. 3a, within a certain range of doses of cellulose (0–25 mg/g materials), the yields of quercetin, luteolin and apigenin increased with increasing doses of cellulase. As the dose of cellulase increased, there was no significant change (*P* < 0.05) in the yields of quercetin, luteolin and apigenin. In addition, the yields of quercetin, luteolin and apigenin were 264.79, 302.45, and 690.07 μg/mg, respectively, when cellulase was not added, which were 55.82, 73.49, and 179.33 μg/mg lower than the 25 mg/g dose of cellulase. The plant cell walls contained mainly cellulose, hemicelluloses and pectin; however, cellulose, a key component of the primary cell wall structure of male inflorescences of *Populus alba × berolinensis*, can be decomposed by cellulase at suitable temperatures and pH values [40]. To minimize cellulase requirements and obtain higher quercetin, luteolin and apigenin yields, a 25 mg/g dose of cellulase would be a better range for optimizing the extraction process of quercetin, luteolin and apigenin.

### 3.3 Effect of the incubation temperature

Optimization of the incubation temperature, which is a significant factor affecting the activity of cellulase used in the process of enzymatic hydrolysis, was necessary to ensure efficient extraction, and the extraction process at different temperatures (30, 40, 50, 60 and 70 °C) was investigated. At an appropriate range of incubation temperatures (30–50 °C), a gradual increase in the quercetin, luteolin and apigenin yields was observed, as shown in Fig. 3b, and the yield reached a maximum plateau at 50 °C. This is because the appropriate temperature is the key to ensuring enzyme activity, thereby promoting enzyme hydrolysis. Instead, the yield gradually decreased with increasing temperature, namely, the incubation temperature was greater than 50 °C. This phenomenon occurred because higher temperatures may reduce enzyme activity or increase the degradation (oxidation) or isomerization of flavonoid compounds and thus decrease the extraction yield [41]. Furthermore, a relatively high incubation temperature reduces the activity of cellulase and thus affects the results. Similarly, Wang reported that arabinoxylan was extracted from wheat bran via ultrasound-assisted enzymatic hydrolysis extraction at different temperatures [42]. In conclusion, incubation temperatures ranging from 50 °C were selected for subsequent experiments.

### 3.4 Effect of incubation time

To investigate the influence of incubation time on the yields of quercetin, luteolin and apigenin, a series of experiments were performed with several incubation times (0, 30, 60, 90, 120, 150, 180 min). From Fig. 3, Fig. 3, a significant increase in quercetin, luteolin and apigenin was observed at various incubation times (0–120 min), beyond which the yield did not obviously change with increasing incubation time. A marked increase in quercetin, luteolin and apigenin was detected at 30 min, and the yield reached a maximum plateau at 120 min. A longer incubation time can provide sufficient time for ensuring that cellulase thoroughly hydrolyses the cell wall. Therefore, 90–150 min is an appropriate incubation time for the following experiments, considering both the time consumption and the yields of quercetin, luteolin and apigenin.

### 3.5 Effect of pH

**Fig. 4:**
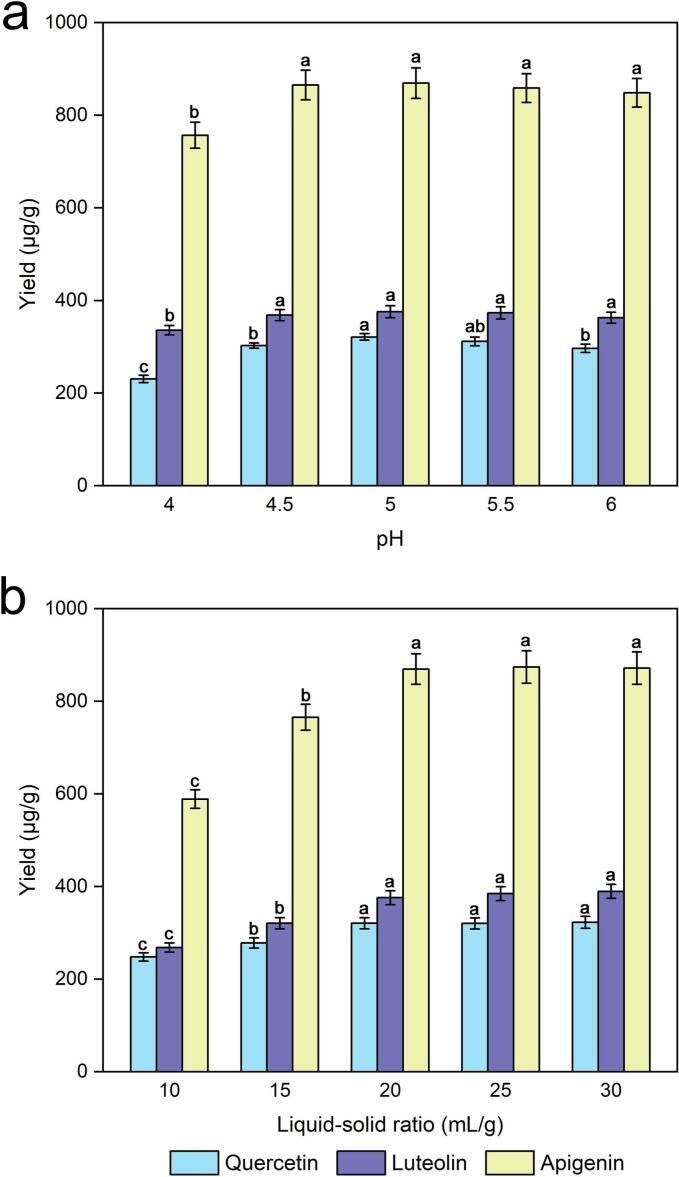
Effects of pH (a) and the liquid‒solid ratio (b) on the extraction yields of quercetin, luteolin and apigenin.

The male inflorescences of *P. alba × berolinensis* powders were also investigated at various pH values (4.0, 4.5, 5, 5.5, and 6). Fig. 4a shows that the yields of quercetin, luteolin and apigenin improved slightly as the pH increased, and the yield reached a maximum when the pH was 5. Nevertheless, a gradual pH increase was associated with a slight decrease in yield. It was speculated that pH could influence the total net charge of enzymes, and the 3D shape of the enzyme changed with denaturation and was unable to catalyze chemical reactions when the enzyme charge was destroyed [43]. Thus, a pH of 5 was a good choice for the following experiments.

### 3.6 Effect of the liquid‒solid ratio

The liquid‒solid ratio, a vital parameter, plays an irreplaceable role in the extraction of quercetin, luteolin and apigenin. Liquid‒solid ratios of 10, 15, 20, 25, and 30 mL/g were used. As shown in Fig. 4b, the yields of quercetin, luteolin and apigenin rapidly increased in the range of 10–15 mL/g liquid‒solid ratio and subsequently increased slightly as the liquid‒solid ratio increased. Similar trends were also reported in previous studies [42,44,45], although the optimal value of the liquid‒solid ratio varied. A lower liquid‒solid ratio might not favoured the movement of the plant cells to the active site of the enzyme and the polysaccharides to the medium under ultrasound irradiation [42]. However, when the liquid-to-solid ratio exceeds a specific threshold, the presence of increased solid particles in the solution leads to inadequate mixing between the sample and solvent, this can cause greater adsorption of the solvent by the solid particles, ultimately resulting in reduced extraction efficiencies [46]. Considering both the consumption of the experimental materials and the yields of quercetin, luteolin and apigenin, a 20 mL/g liquid‒solid ratio was adopted as the ideal condition for extraction.

### 3.7 Effect of ultrasonic irradiation power on the incubation process

**Fig. 5:**
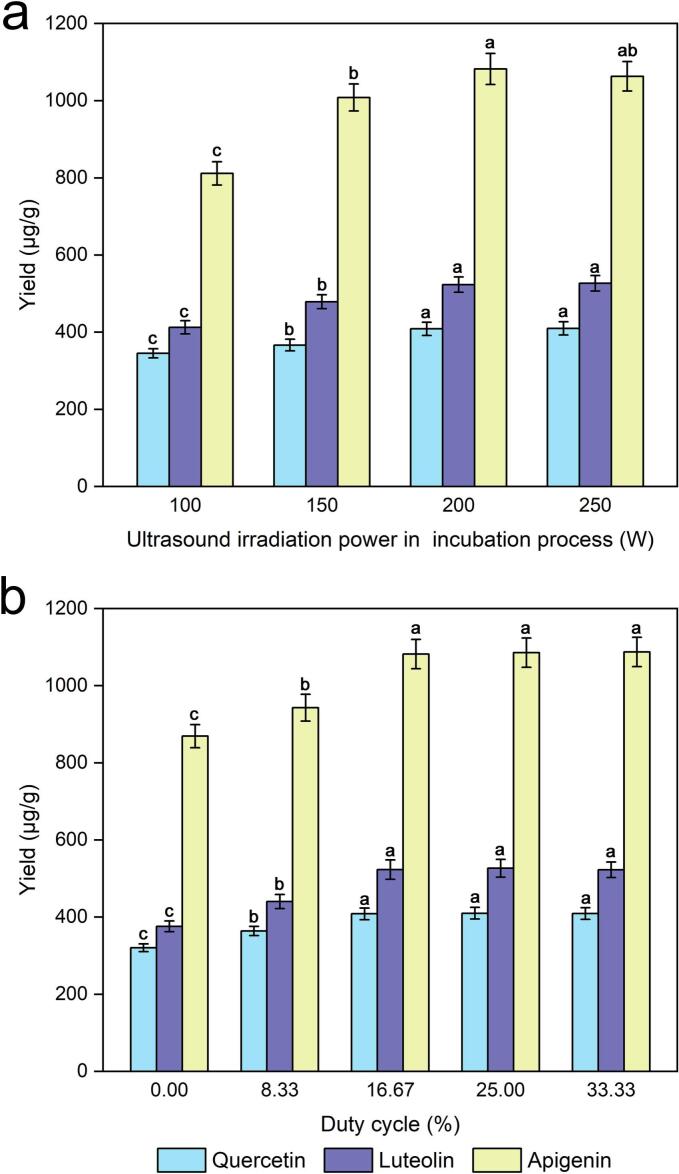
Effect of ultrasound irradiation power during the incubation process (a) and duty cycle (b) on the extraction yields of quercetin, luteolin and apigenin.

The ultrasonic irradiation power used in the incubation process is also a critical factor that affects the enzymatic hydrolysis process, and several tests have been performed with different ultrasonic irradiation powers (100, 150, 200, 250 W). As shown in Fig. 5a, all yields were greater than the other yields when the ultrasonic irradiation power was greater than 200 W. A higher ultrasonic irradiation power was beneficial for increasing the degradation of *P. alba × berolinensis* cell walls, which may be the main reason for the higher quercetin, luteolin and apigenin extraction rates. Therefore, 200 W of ultrasonic irradiation power was selected for the incubation process, which provided enough energy to ensure high extraction efficiency.

### 3.8 Effect of the duty cycle

To investigate the influence of the duty of the cycle effect, some experiments were conducted with different duty numbers of cycles (0 %, 8.33 %, 16.77 %, 25.00 %, and 33.33 %). As shown in Fig. 5, Fig. 5, a significant increase was observed in the enzymatic hydrolysis process during the cycle treatment compared with the control treatment. There was no obvious change in the yields of quercetin, luteolin or apigenin as the number of cycles increased from 16.67 % to 33.33 %. This phenomenon may be attributed to the fact that the duty of the cycle increases the rate of enzymatic hydrolysis. Furthermore, ultrasound-assisted enzymatic hydrolysis has been shown to significantly improve the mass transfer efficiency of enzyme macromolecules between the solid surface and the reaction medium. Finally, a cycle duty of 16.67 % was considered the optimal value for the following experiments.

### 3.9 Effect of the ultrasonic irradiation power on the extraction process

**Fig. 6:**
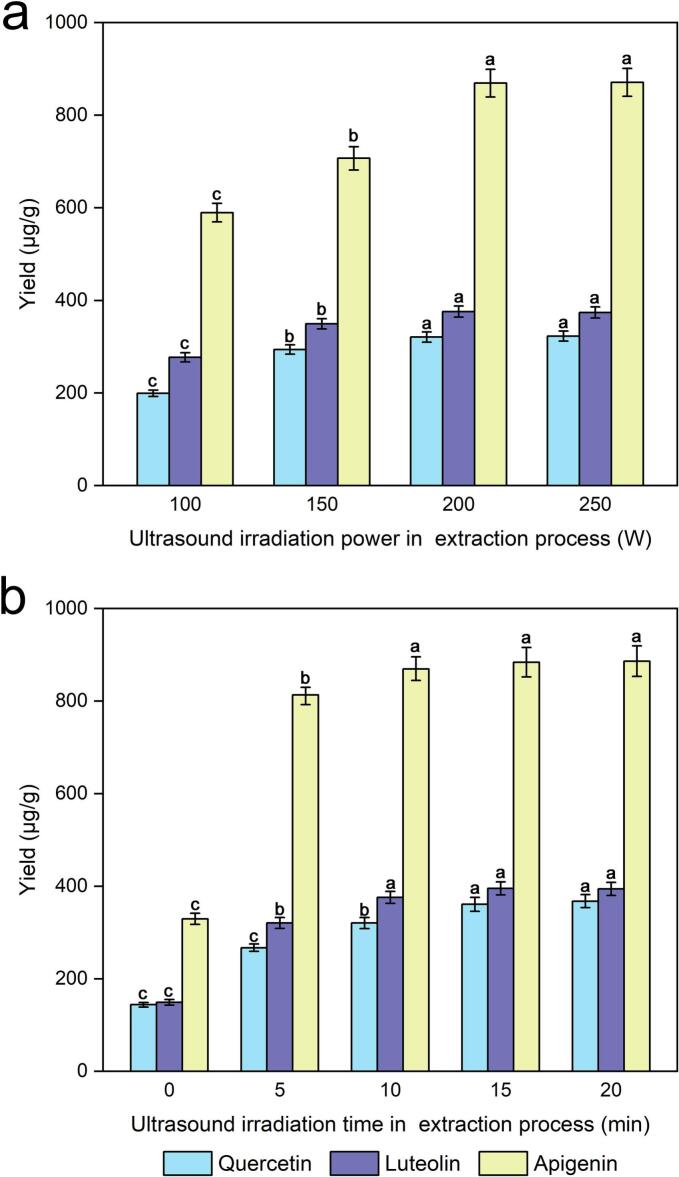
Effect of ultrasonic irradiation power during the extraction process (a) and ultrasonic irradiation time during the extraction process (b) on the extraction yields of quercetin, luteolin and apigenin.

Ultrasound irradiation power, a key factor affecting the yield of quercetin, luteolin and apigenin to a large extent, was investigated via extraction (0, 100, 150, 200 and 250 W). Fig. 6a clearly shows that different degrees of ultrasonic irradiation power were more conducive to the extraction efficiency of quercetin, luteolin and apigenin than the blank group was. With increasing ultrasonic irradiation power in the extraction process from 100 to 250 W, the yields were correspondingly increased. The highest yields of quercetin, luteolin and apigenin were achieved when the ultrasound irradiation power was 200 W, and there was no obvious change in yield when the ultrasonic irradiation power was increased beyond 200 W. This result was attributed to ultrasonic treatment during the extraction process of quercetin, luteolin and apigenin resulting in cavitation and increased diffusion of the solvent and target ingredients, which may affect the extraction efficiency related to the amount of energy generated by the ultrasonic power source [39]. Thus, 200 W of ultrasonic irradiation power during the extraction process was used for further optimization tests.

### 3.10 Effect of ultrasonic irradiation time on the extraction process

As shown in Fig. 6b, the yields of quercetin, luteolin and apigenin increased significantly during the first 10 min of the ultrasonic irradiation process and subsequently improved with increasing ultrasonic irradiation time, but the subsequent increase rate was lower than that during the first ten minutes. A previous study indicated that short-term ultrasound exposure may increase enzyme activity [47]. In addition, the activities of the enzymes improved, and the diffusion of particles or external solvents into the cells improved with increasing ultrasonic irradiation time during the extraction process, which may have facilitated the release of quercetin, luteolin and apigenin from plant cells; consequently, a higher yield was obtained [35,39]. The experimental results revealed that the optimal ultrasound irradiation time in the extraction process for maximum yields of quercetin, luteolin and apigenin should be 15 min for the experiments.

### 3.11 Parameter optimization via response surface methodology (RSM)

To optimize the effects of three process parameters (dose of cellulase, incubation temperature, incubation time) on the yields of quercetin, luteolin and apigenin, a Box–Behnken design (BBD) combined with RSM was used in the present work. A cellulase dose of 25 mg/g, an incubation temperature of 50 °C, and an incubation time of 120 min were selected as the central conditions of the BBD. BBD for the experimental values and predicted values for yields of quercetin, luteolin and apigenin are presented in Table 1. Table 1 shows that the maximum yields of quercetin (428.89 μg/g), luteolin (549.41 μg/g) and apigenin (1136.23 μg/g) were recorded when the experimental parameters of cellulase were 25 mg/g, the incubation temperature was 50 °C, and the incubation time was 120 min. The lowest yields of quercetin (320.78 μg/g), luteolin (448.031 μg/g) and apigenin (952.54 μg/g) were observed in different tests. Moreover, the yields of quercetin (424.89 μg/g), luteolin (547.30 μg/g) and apigenin (126.21 μg/g) were also high, with 40 mg/g cellulase, an incubation temperature of 50 °C, and an incubation time of 150 min without much variation compared with the highest yield. This might have contributed to the similar stabilities of the enzymes within the tested temperature and time ranges [42].

**Table 1:** Box–Behnken design (BBD) for the experimental values and predicted values for yields of quercetin, luteolin and apigenin.

Run	Factor *X_1_*	Factor *X_2_*	Factor *X_3_*	Yield (μg/g)		
	Dose of cellulase (mg/g）	Incubation temperature (°C)	Incubation time (min)	Quercetin	Luteolin	Apigenin
1	10	40	120	320.78	500.84	966.56
2	40	40	120	370.83	498.72	1009.31
3	10	60	120	390.85	448.03	959.88
4	40	60	120	405.54	463.52	1108.17
5	10	50	90	352.81	523.36	1014.66
6	40	50	90	400.20	517.73	1092.81
7	10	50	150	420.22	543.08	1110.85
8	40	50	150	424.89	547.30	1126.21
9	25	40	90	328.12	514.21	952.54
10	25	60	90	395.52	452.26	1007.98
11	25	40	150	394.19	540.97	1043.38
12	25	60	150	400.86	480.42	1107.51
13	25	50	120	421.55	519.14	1136.23
14	25	50	120	428.89	548.71	1135.56
15	25	50	120	428.89	549.41	1113.52
16	25	50	120	428.23	548.01	1136.23
17	25	50	120	428.89	548.01	1135.56

The quadratic model’s regression coefficients and variance analysis, as determined by the BBD for extracting quercetin, luteolin and apigenin, are summarized in Table 2. The predicted model *F* values of *Y_1_, Y_2_ and Y_3_* were 62.57, 16.94, and 26.38, respectively, and their *P* values were all less than 0.05, which indicated that the model was significant. Lack of fit is an important index used to evaluate the reliability of the equation. The *P* values of *Y_1_, Y_2_, and Y_3_* for lack of fit were 0.0617, 0.7953, and 0.0620 (>0.05), respectively, which implied that this regression equation could effectively describe the relationships between different factors and response values. The credibility analysis of the regression equations revealed that the coefficient of determination (*R^2^*) values for the predicted models of *Y_1_, Y_2_, and Y_3_* were 0.9877, 0.9561, and 0.9714, respectively, whereas the adjusted determination coefficient (Adjust. The *R^2^* values for the predicted models of *Y_1_, Y_2_, and Y_3_* were 0.9719, 0.8997, and 0.9345, respectively, which indicated that the model was very suitable for processing the experimental results and that the experimental and predicted values had high degrees of correlation. The signal‒to-noise ratios (adesq precisions) of *Y_1_, Y_2_, and Y_3_* were 23.67, 12.20, and 13.45, respectively, which were greater than 4.0, indicating that the model had a good predictive effect. The results collectively demonstrated that the predicted model provided accurate predictions across the experimental variable ranges.

**Table 2:** Estimated regression coefficients and analysis of variance for the response surface quadratic model determined from BBD for quercetin, luteolin and apigenin extraction.

	Degree of freedom	*Y_1_*		*Y_2_*		*Y_3_*	
		*F*-value	*P*-value	*F*-value	*P*-value	*F*-value	*P*-value
Model	9	62.57	< 0.0001*	16.94	0.0006*	26.38	0.0001*
*X_1_*	1	50.47	0.0002*	0.14	0.7154	35.32	0.0006*
*X_2_*	1	118.45	< 0.0001*	44.55	0.0003*	18.30	0.0037*
*X_3_*	1	99.22	< 0.0001*	10.90	0.0131*	39.14	0.0004*
*X_1_X_2_*	1	9.20	0.0190*	0.62	0.4556	9.25	0.0188*
*X_1_X_3_*	1	13.71	0.0076*	0.20	0.6717	2.40	0.1656
*X_2_X_3_*	1	26.97	0.0013*	0.00	0.9478	0.06	0.8106
*X_1_*^2^	1	38.83	0.0004*	7.09	0.0324*	14.57	0.0066*
*X_2_*^2^	1	175.42	< 0.0001*	86.07	< 0.0001*	106.96	< 0.0001*
*X_3_*^2^	1	12.61	0.0093*	0.75	0.4155	3.36	0.1093
Residual	7						
Lack of fit	3	5.77	0.0617	0.35	0.7953	5.76	0.0620
Pure error	4						
Cor total	16						

Credibility analysis of the regression equations	Index mark	Standard deviation	Mean	CV %	Press	*R^2^*	Adjust *R^2^*	Predicted *R^2^*	Adequacy precision
	*Y_1_*	5.81	396.49	1.46	3138	0.9877	0.9719	0.8368	23.67
	*Y_2_*	11.14	514.32	2.17	3943	0.9561	0.8997	0.8010	12.20
	*Y_3_*	17.45	1067.39	1.63	28,318	0.9714	0.9345	0.6195	13.45

* Significant at *P* < 0.05.

**Fig. 7:**
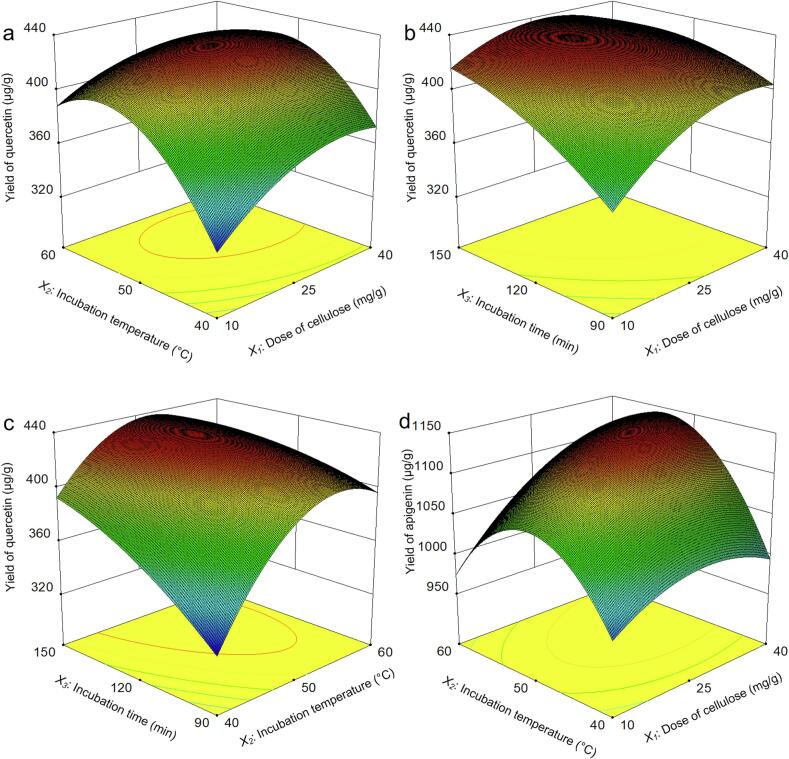
Optimization of yields of quercetin, luteolin and apigenin via Box–Behnken design (BBD). Interaction effect of the cellulase dose and incubation temperature on the yield of quercetin (a); interaction effect of the cellulase dose and incubation time on the yield of quercetin (b); interaction effect of the incubation temperature and incubation time on the yield of quercetin (c); and interaction effect of the cellulase dose and incubation temperature on the yield of apigenin (d).

The response surfaces were plotted by Design Expert software, which demonstrated the influences of three parameters (dose of cellulase, incubation temperature, incubation time) and their interactions on the yields of quercetin, luteolin and apigenin. Fig. 7a The interaction effect of the dose of cellulase (*X_1_*) and incubation temperature (*X_2_*) on the yield of quercetin revealed that these two factors strongly influence the yield of quercetin and the curvature of the response surface. With increasing doses of cellulase and incubation temperatures, the enzyme reaction rate increased, and the yield of quercetin increased significantly. A slight decrease in the yield of quercetin was subsequently observed when the dose of cellulase and incubation temperature increased further. Increasing the temperature may damage the tertiary structure of enzymes, eventually leading to enzyme denaturation, thus decreasing the yield of quercetin [42]. From the 3D response surface of Fig. 7b, the effects of the interaction of the dose of cellulase (*X_1_*) and incubation time (*X_3_*) on the yield of quercetin are shown. The yield of quercetin first improved and then remained constant as the dose of cellulase and incubation time increased. Although a higher dose of cellulase and longer incubation time improved the yield of quercetin, the dose of cellulase and incubation time could not be increased indefinitely. Fig. 7c shows the interaction effect of the incubation temperature (*X_2_*) and incubation time (*X_3_*) on the yield of quercetin, from which the yield gradually increased as the incubation temperature increased and the incubation time increased, whereas a significant reduction in the quercetin yield was observed as the experiment progressed. Fig. 7d shows the interaction effect of the dose of cellulase (*X_1_*) and incubation temperature (*X_2_*) on the yield of apigenin, which had the same variation tendency as that shown in Fig. 7a, but the degree of variation in the yield of apigenin in Fig. 7d was greater than that in the yield of quercetin in Fig. 7a.

Considering the yields of quercetin, luteolin and apigenin, the optimum conditions predicted by the software were as follows: 40 mg/g of cellulase, 49.31 °C of incubation temperature, and 147.75 min incubation time. However, for practical convenience, the actual parameter conditions are as follows: 40 mg/g of cellulase, 50 °C incubation temperature, and 148 min incubation time. Moreover, the predicted yields of quercetin, luteolin and apigenin via software were 424.33 μg/g, 549.25 μg/g and 1141.24 μg/g, respectively. Under these conditions, three sets of verification tests were carried out, and the quercetin, luteolin and apigenin yields were 425.20, 550.33, and 1140.79 μg/g, respectively.

### 3.12 Model adequacy survey

Model adequacy was measured by some diagnostic plots. Three diagnostic plots, namely the actual responses versus the predicted responses, the normal plot of residuals and internally Studentized residuals versus run number, are shown in Fig. S1. Fig. S3a, b and c showed that all reasonably aligned points were distributed and rotated around a straight line, which showed ac high degree of matching between the actual and predicted values obtained by the model. As we can see from Fig. S3d, e and f, all points in the normal plot of residuals were close to contact with the straight line, meaning that the model of quercetin, luteolin and apigenin were stable and accurate and complied with a normal distribution. The plot of internally Studentized residuals versus run number was employed as shown in Fig. S3g, h and i. All data points exhibited a random scatter distribution within certain limits (±3), which showed a good fit of the responses to the developed model. These results repeating back and forth ensured that the targets are extracted efficiently.

### 3.13 Comparison with references and conventional methods

The flavonoids extracted by UACHEE were compared with those extracted via conventional methods of heat reflux extraction (HRE) [48]. Table 3 shows that the yields of quercetin, luteolin and apigenin extracted by UACHEE were higher than those extracted by HRE. For the UACHEE method without cellulase hydrolysis pretreatment, the extracted solvents of pure water had lower yields of 247.27, 353.46 and 377.19 μg/g than did the 60 % volume fraction ethanol. The extraction rate of the target components from the raw materials treated with cellulase was better than that of the untreated materials, which was speculated that the enzyme can destroy the plant cell wall and facilitate the dissolution of the target components [49]. In terms of the HRE method, regardless of whether enzyme pretreatment was carried out with pure water or a 60 % volume fraction of ethanol, the yields of its target ingredients were relatively low, which took a long time. On the basis of the above discussion, cellulase hydrolysis pretreatment combined with ultrasonic-assisted extraction resulted in increased extraction yields of quercetin, luteolin and apigenin.

**Table 3:** Comparison of the yields of quercetin, luteolin and apigenin by different methods.

Method	Pretreatment	Solvent	Temperature (℃)	Extraction time (min)	Yield (μg/g)		
					Quercetin	Luteolin	Apigenin
Ultrasonic-assisted extraction (UAE)	/	pure water	Room temperature	30	138.15 ± 5.75^g^	156.77 ± 4.48^f^	520.36 ± 10.39^e^
	/	60 % volume fraction ethanol	Room temperature	30	385.62 ± 12.21^c^	510.23 ± 9.96^b^	897.55 ± 25.64^c^
	cellulase hydrolysis	pure water	Room temperature	120	198.23 ± 7.62^e^	201.33 ± 6.97^d^	630.56 ± 14.26^d^
	cellulase hydrolysis	60 % volume fraction ethanol	Room temperature	120	428.68 ± 10.14^a^	550.21 ± 10.14^a^	1136.20 ± 32.38^a^
Heat reflux extraction (HRE)	/	pure water	85	120	/	/	/
	/	60 % volume fraction ethanol	85	120	295.41 ± 10.34^d^	456.24 ± 8.55^c^	886.98 ± 24.38^c^
	cellulase hydrolysis	pure water	85	240	186.38 ± 6.30^f^	180.11 ± 5.96^e^	621.09 ± 12.51^d^
	cellulase hydrolysis	60 % volume fraction ethanol	85	240	412.56 ± 8.27^b^	549.65 ± 11.21^a^	1025.69 ± 30.96^b^

Values in each column with different letters are significantly different (p *<* 0.05).

### 3.14 Method validation

The limits of detection (LODs) for quercetin, luteolin and apigenin were determined to be 0.0385, 0.0783, and 0.0748 mg/mL, respectively, while their corresponding limits of quantification (LOQs) were 0.1177, 0.2374, mg/mL and 0.2267 mg/mL, respectively. Stability and recovery studies demonstrated that the UACHEE extraction method coupled with HPLC analysis achieved optimal recovery rates for both analytes.

The stability and recovery studies of quercetin, luteolin and apigenin standards were evaluated under the following UACHEE conditions: ethanol volume fraction of 70 %, 40 mg/g of dose of cellulase, 50 °C of incubation temperature, 148 min of incubation time, pH of 5, 20 mL/g of liquid‒solid ratio, duty cycle of 16.67 %, 200 W of ultrasound irradiation power in the incubation process, 200 W of ultrasound irradiation power in the extraction process, and 15 min of ultrasound irradiation time in the extraction process. Table S1 showed that the average recovery of the standard solution of quercetin, luteolin and apigenin was 99.03 %, 98.56 % and 98.67 %, the recovery of the recovered concentration of quercetin, luteolin and apigenin after 7 days were 97.22 %, 97.56 % and 96.84 %, respectively. The results confirmed that no thermal isomerization or degradation occurred under the predicted operational parameters.

Method accuracy was assessed by spiking male inflorescences of *P. alba × berolinensis* samples with quercetin, luteolin and apigenin standard solutions at low, medium and high concentrations, followed by HPLC analysis. The measured quercetin, luteolin and apigenin contents were used to calculate recovery rates, which were respectively 98.11 %, 98.14 % and 98.42 %, confirming the accuracy of the method. Method precision was confirmed by intraday and interday assays (RSD < 2 %). Detailed precision data are provided in Table S1, which supported the reproducibility of the method.

### 3.15 Mechanistic exploration of the UACHEE process

#### 3.15.1 SEM analysis of untreated and treated samples

**Fig. 8:**
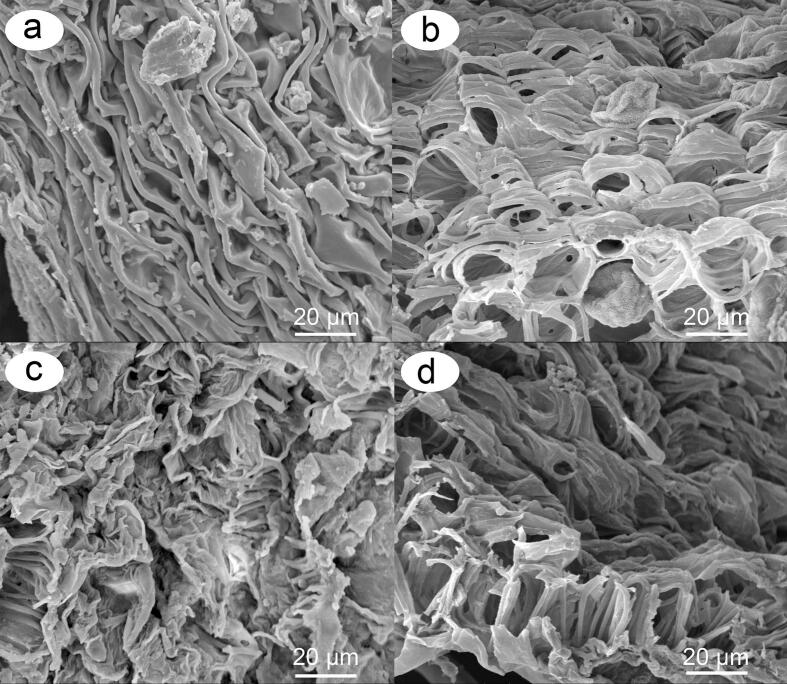
SEM images of male inflorescences of *Populus alba* × *berolinensis* before and after treatment. Raw sample (a); ultrasonic treatment (ultrasonic irradiation power: 200 W, ultrasound irradiation time: 30 min) (b); cellulase hydrolysis treatment (incubation temperature: 50 °C, incubation time: 120 min) (c); and ultrasound-assisted cellulase hydrolysis treatment (ultrasound irradiation power: 200 W; ultrasound irradiation time: 30 min; incubation temperature: 50 °C; incubation time: 120 min) (d).

To assess the effectiveness of enzymatic hydrolysis and ultrasonic extraction on the microscopic structural changes in the cell wall, the surface morphology of the untreated and enzymatically treated samples of male inflorescences of *Populus alba × berolinensis* was analyzed via scanning electron microscopy (SEM). Fig. 8a shows that the untreated sample presented a nonporous, tight, compact and complete microscopic structure with no signs of pitting or damage to the cell wall. However, Fig. 8b shows that the material treated with ultrasound (ultrasonic time: 30 min, ultrasonic power: 200 W) had a loose and matte surface, and some filamentous structures could be observed. Compared with Fig. 8b, Fig. 8c shows some significant changes in that some smaller fragments were produced from samples treated by enzymatic hydrolysis (incubation temperature: 50 °C, incubation time: 2 h). Compared with pure enzymatic hydrolysis and ultrasonic treatment, ultrasonic-assisted enzymatic hydrolysis treatment (incubation temperature: 50 °C; incubation time: 2 h; ultrasonication time: 30 min; ultrasonic power: 200 W) caused much more porous structures and cell wall debris, as shown in Fig. 8d, which increased the contact area of the raw material with cellulose [50] and then helped cellulase penetration into the interior of the plant tissue structure. As a result, the efficiency of enzymatic hydrolysis was enhanced.

#### 3.15.2 Fourier transform infrared (FTIR) spectroscopy analysis of untreated and treated samples

**Fig. 9:**
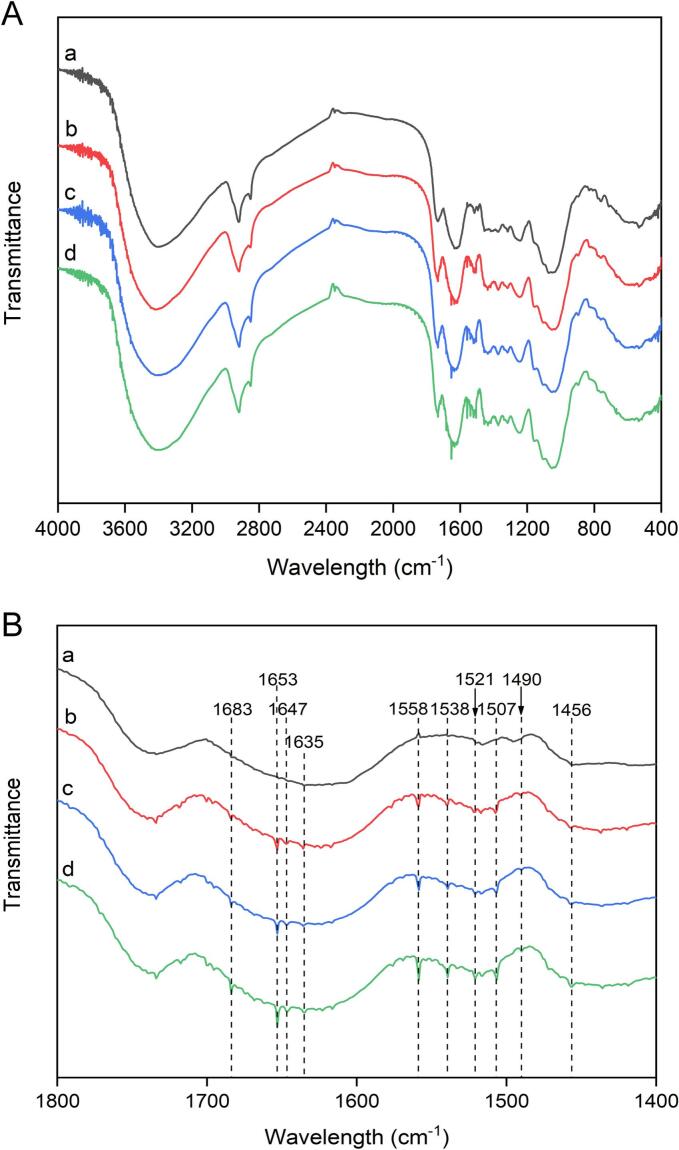
FTIR images of male inflorescences of *Populus alba* × *berolinensis* before and after treatment. Raw sample (a); ultrasonic treatment (ultrasonic irradiation power: 200 W, ultrasound irradiation time: 30 min) (b); cellulase hydrolysis treatment (incubation temperature: 50 °C, incubation time: 120 min) (c); and ultrasound-assisted cellulase hydrolysis treatment (ultrasound irradiation power: 200 W; ultrasound irradiation time: 30 min; incubation temperature: 50 °C; incubation time: 120 min) (d).

The FTIR spectra of the untreated, enzymatic hydrolysis-treated and ultrasonic extraction-treated samples are shown in Fig. 9a, and the wavelengths were in the range of 4000–400 cm^−1^. The signal peaks located in the range from 1800–1400 cm^−1^ are shown in Fig. 9b. Fig. 9b shows that the absorption peaks of the untreated and treated samples were significantly different. Carboxylic acids usually exist in the form of two-molecule associations due to hydrogen bonding, and their absorption peaks appear at approximately 1725–1700 cm^−1^. The peak near 1700 cm^−1^ easily identified via infrared spectroscopy was the C=O group stretching vibration of ester carbon [51]. Fig. 9c shows that the region from 1680 to 1620 cm^−1^ presented obvious scaling changes, which were assigned to the C=O stretching vibration. In particular, the bands at 1680 cm^−1^, 1653 cm^−1^, 1647 cm^−1^, 1635 cm^−1^ and 1620 cm^−1^ exhibited different degrees of stretching vibration in the untreated samples compared with the treated samples. In particular, the absorption peak at approximately 1620 cm^−1^ corresponds to the C=O stretching vibration of free carboxyl groups [50]. The peak at 1460 ± 10 cm^−1^ was attributed predominantly to asymmetrical CH_3_ stretching and CH_2_ vibrations in most organic compounds [52]. SEM combined with FTIR revealed that the microscopic structure of the plant cell wall was destroyed by the process of ultrasound-assisted enzymatic hydrolysis.

#### 3.15.3 X-ray diffraction (XRD) analysis

**Fig. 10:**
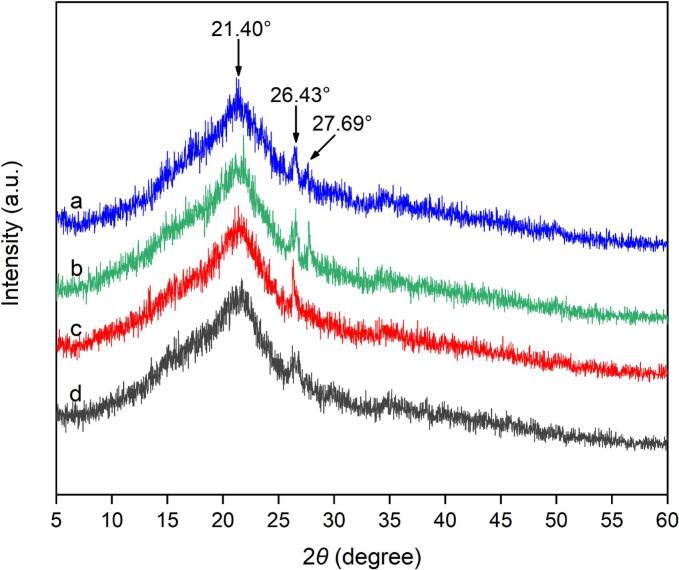
XRD images of male inflorescences of *Populus alba* × *berolinensis* before and after treatment. Raw sample (a); ultrasonic treatment (ultrasonic irradiation power: 200 W, ultrasound irradiation time: 30 min) (b); cellulase hydrolysis treatment (incubation temperature: 50 °C, incubation time: 120 min) (c); and ultrasound-assisted cellulase hydrolysis treatment (ultrasound irradiation power: 200 W; ultrasound irradiation time: 30 min; incubation temperature: 50 °C; incubation time: 120 min) (d).

The plant cell wall, a dynamic and recalcitrant matrix, primarily consists of cellulose, hemicelluloses, pectins, enzymes and structural proteins [53] and is a complex secondary cell wall matrix [54]. It has been reported that reducing the crystallinity of cellulose is beneficial for improving the enzymatic hydrolysis efficiency. The results for the untreated and treated samples are presented in Fig. 10. As shown in Fig. 10, Fig. 10, three representative diffraction peaks corresponding to the characteristics of sharpness at 2*θ* values of 21.40°, 26.43° and 27.69° were observed. **H**owever, as shown in Fig. 10, Fig. 10, after cellulase hydrolysis treatment and ultrasound-assisted cellulase hydrolysis treatment, only two representative diffraction peaks corresponding to sharpness at 2*θ* values of 21.40° and 26.43° were observed. This phenomenon revealed that the crystallinity of the raw material changed in the presence of cellulase, which may be attributed to a chemical change in which cellulase enzymatically degrades the components of plant cell walls.

#### 3.15.4 Possible mechanism of extraction of quercetin, luteolin and apigenin by UACHEE

Compared with other pressing methods, the prominent advantage of the use of UACHEE to extract target components is the dual role of the cavitation phenomena of ultrasonic treatment and cellulase hydrolysis, and the mechanism diagram was presented in Fig. S4.

Cavitation is a mechanical effect generated by pressure changes in a liquid medium under the action of ultrasonic irradiation, and the cavitation phenomenon is subjected to different stages, including the formation, growth, collapse or implosion of bubbles [55]. When the ultrasound system was working, the sinusoidal ultrasonic waves were converted to mechanical vibrations, which led to compression and rarefaction in the liquid medium. The local pressure changes produce tiny gas bubbles. When the bubbles grow to a critical size, they implode and thus form a cavitation phenomenon, which is the most important effect of high-power ultrasound [56]. Moreover, the ultrasonic waves are transformed into thermal energy, resulting in a thermal effect, which accelerates the dissolution of the inner components of the target into the extraction solvent. Several mechanisms, including erosion, capillarity or sonoporation, have been shown to affect the process of ultrasonic-assisted extraction, thereby facilitating the breakdown of plant cell walls and the subsequent release and solubilization of target compounds into the solvent [35].

On the other hand, cellulose is the main component of plant cell walls, and cellulase hydrolysis can fracture the *β*-glucoside bonds of cellulose, which could be effective in modifying the structure of plant cell walls and the dissolution of flavonoids [57]. The SEM, FTIR and XRD results also clarified the possible extraction mechanism of UACHEE to some extent. Furthermore, compared with conventional extraction techniques, ultrasound-enhanced enzymatic hydrolysis has shown superior extraction efficiency, achieving increased yields at reduced temperatures and shorter processing times.

## 4 Conclusions

Combination of ultrasound-assisted cellulase hydrolysis and ethanol extraction, as an efficient and environmentally friendly strategy, has been evaluated for the extraction of quercetin, luteolin and apigenin from male inflorescences of *P. alba × berolinensis*. A series of single experiments and three-variable, three-level experiments with a Box–Behnken design based on RSM were performed to optimize the yields of quercetin, luteolin and apigenin. Furthermore, under the optimized conditions, three sets of verification tests were carried out, and the extraction yields of quercetin, luteolin and apigenin were 425.200 μg/g, 550.332 μg/g, and 1140.786 μg/g, respectively. Compared with other UAE and HRE methods, the UACHEE process improved the yields of three flavonoids. In addition, to clarify the advantages of this method in extracting target components, we explored the mechanism of the UACHEE process in detail. Ultrasonic-assisted enzymatic hydrolysis and ethanol extraction can improve extraction efficiency at reduced temperatures and shorter durations, which may be more powerful and efficient methods for extracting active ingredients. Future work could explore scale-up feasibility or solvent recycling efficiency.

## CRediT authorship contribution statement

**Ru Zhao:** Writing – original draft, Validation, Software, Methodology, Funding acquisition, Formal analysis, Data curation. **Xiaoli Li:** Validation, Software, Methodology, Investigation. **Xiuqi Wu:** Validation, Software, Formal analysis. **Yulong Wu:** Resources, Methodology, Funding acquisition. **Ning Tang:** Validation, Software, Resources. **Chen Xu:** Validation, Resources, Methodology. **Tingli Liu:** Writing – original draft, Resources, Project administration, Funding acquisition, Data curation. **Ailing Ben:** Validation, Resources, Project administration, Funding acquisition.

## Declaration of competing interest

The authors declare that they have no known competing financial interests or personal relationships that could have appeared to influence the work reported in this paper.
