# Supplementary material for: Optimization, comparison and mechanism of ultrasound-assisted cellulase hydrolysis and ethanol extraction of quercetin, luteolin and apigenin from male inflorescences of Populus alba × berolinensis
Source: Ultrason Sonochem. 2025 Oct 22;123:107645. doi: 10.1016/j.ultsonch.2025.107645 (PMC12630045; doi:10.1016/j.ultsonch.2025.107645)
Supplement: Supplementary Data 1 [file mmc1.docx]

Process optimization, method comparison and mechanism exploration of ultrasound-assisted cellulase hydrolysis and ethanol extraction of quercetin, luteolin and apigenin from male inflorescences of *Populus alba × berolinensis*

Ru Zhao^a^, Xiaoli Li^a^, Xiuqi Wu^a,b^, Yulong Wu^a^, Ning Tang^a^, Chen Xu^a^, Tingli Liu^a*^, Ailing Ben^a*^

^a^Nanjing Engineering Research Center for Peanut Genetic Engineering Breeding and Industrialization, School of Food Science, Nanjing Xiaozhuang University, Nanjing 211171, China;

^b^Department of Biology, University of North Carolina at Chapel Hill, Chapel Hill, NC, 27599-3280, USA.

* Corresponding author E-mail: liutingli@njxzc.edu.cn; benailing@njxzc.edu.cn

1. Introduction

Research has reported that the male inflorescences of *Flos populi* contain glycosides, cardiotonics, flavanoids and phenols^[7,8]^. There are further studies on the adsorption and desorption characteristics of macroporous resins for quercetin, luteolin and apigenin from *Flos populi*^[9]^. We also extracted some flavonoid ingredients, such as quercetin, luteolin, and apigenin from the male inflorescence of *P. alba* × *berolinensis*. The structures of the three flavonoids are presented in Figure S1. Quercetin has been reported to have various therapeutic applications, such as anticancer, anti-inflammatory, antiobesity, arthritis, allergy and asthma treatments^[10,11]^. Further investigations indicated that quercetin also has other applications for inducing visual monitoring of fish spoilage^[12]^, regulation of glucolipid metabolism disorders^[13]^, cell protection via erythropoietin^[14]^, cutaneous wound healing^[10]^, and anti-inflammatory and analgesic drugs^[15]^. Recently, luteolin, a Chinese herb found in many fruits and green plants^[16]^, has caused widespread concern due to its pharmacological effects, such as anticancer potential^[17]^, hepatoprotective^[18]^, reducing inflammation^[19]^, neuroprotection^[20]^, antioxidant and antitumor effects^[21]^. Some studies have indicated that apigenin has various pharmacological activities, including anticancer^[22]^, anti-inflammatory and antioxidant activities^[11]^. Quercetin, luteolin and apigenin, as natural ingredients, have become popular research topics.


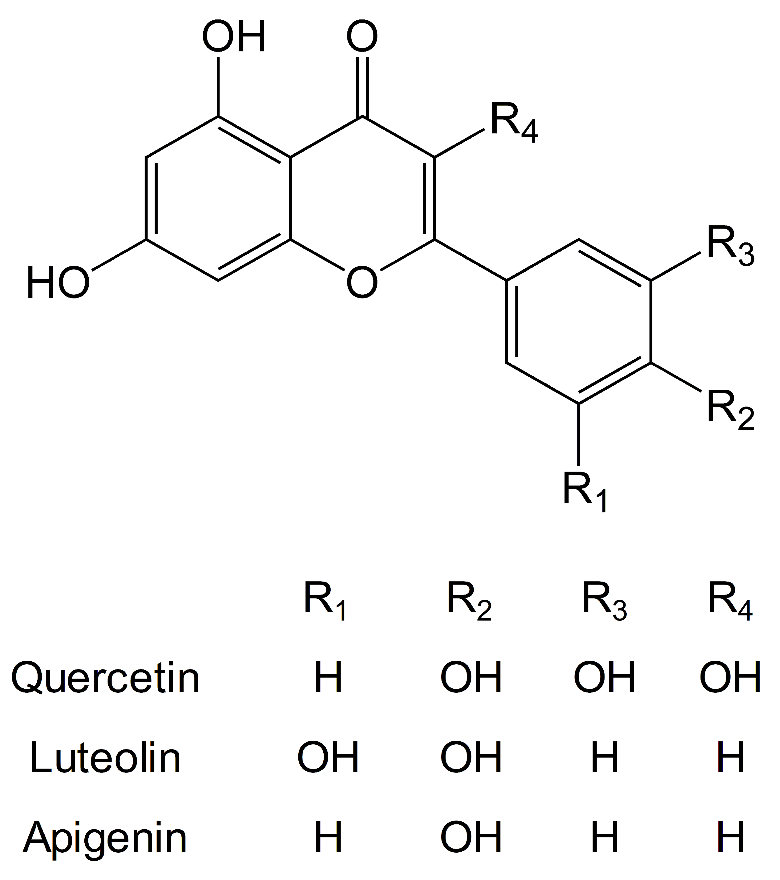


Figure S1. The molecular structures of quercetin, luteolin and apigenin.

2.1. Raw materials and reagents

Male inflorescences of *P. alba × berolinensis* were obtained in April 2023 from Harbin, Heilongjiang Province, Northeast China, and were authenticated by Prof. Ailing Ben from Nanjing Xiaozhuang University, China. The appearance of male inflorescences of *P. alba × berolinensis* is shown in Figure S2. Before the experiments were started, the raw materials were crushed with a disintegrator and then sieved with a 60 mesh sieve. The crushed sample was preserved in an airtight container with a cool and dry environment for all the experiments. The moisture content of the male inflorescences of *P. alba × berolinensis* was measured as 6.59%.


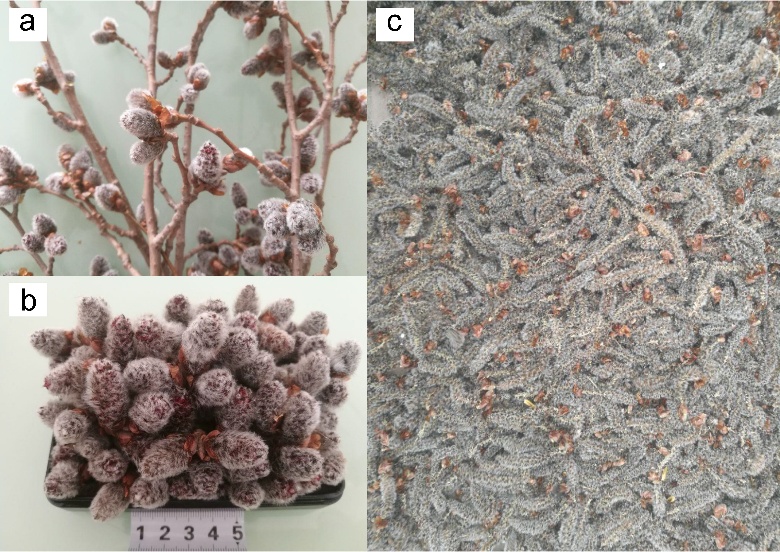


Figure S2. Physical maps of the male inflorescences of *Populus alba × berolinensis*.

3.12. Model adequacy survey

Model adequacy was measured by some diagnostic plots. Three diagnostic plots, namely the actual responses versus the predicted responses, the normal plot of residuals and internally Studentized residuals versus run number, are shown in Figure S1. Figure S3a, b and c showed that all reasonably aligned points were distributed and rotated around a straight line, which showed ac high degree of matching between the actual and predicted values obtained by the model. As we can see from Figure S3d, e and f, all points in the normal plot of residuals were close to contact with the straight line, meaning that the model of quercetin, luteolin and apigenin were stable and accurate and complied with a normal distribution. The plot of internally Studentized residuals versus run number was employed as shown in Figure S3g, h and i. All data points exhibited a random scatter distribution within certain limits (±3), which showed a good fit of the responses to the developed model. These results repeating back and forth ensured that the targets are extracted efficiently.


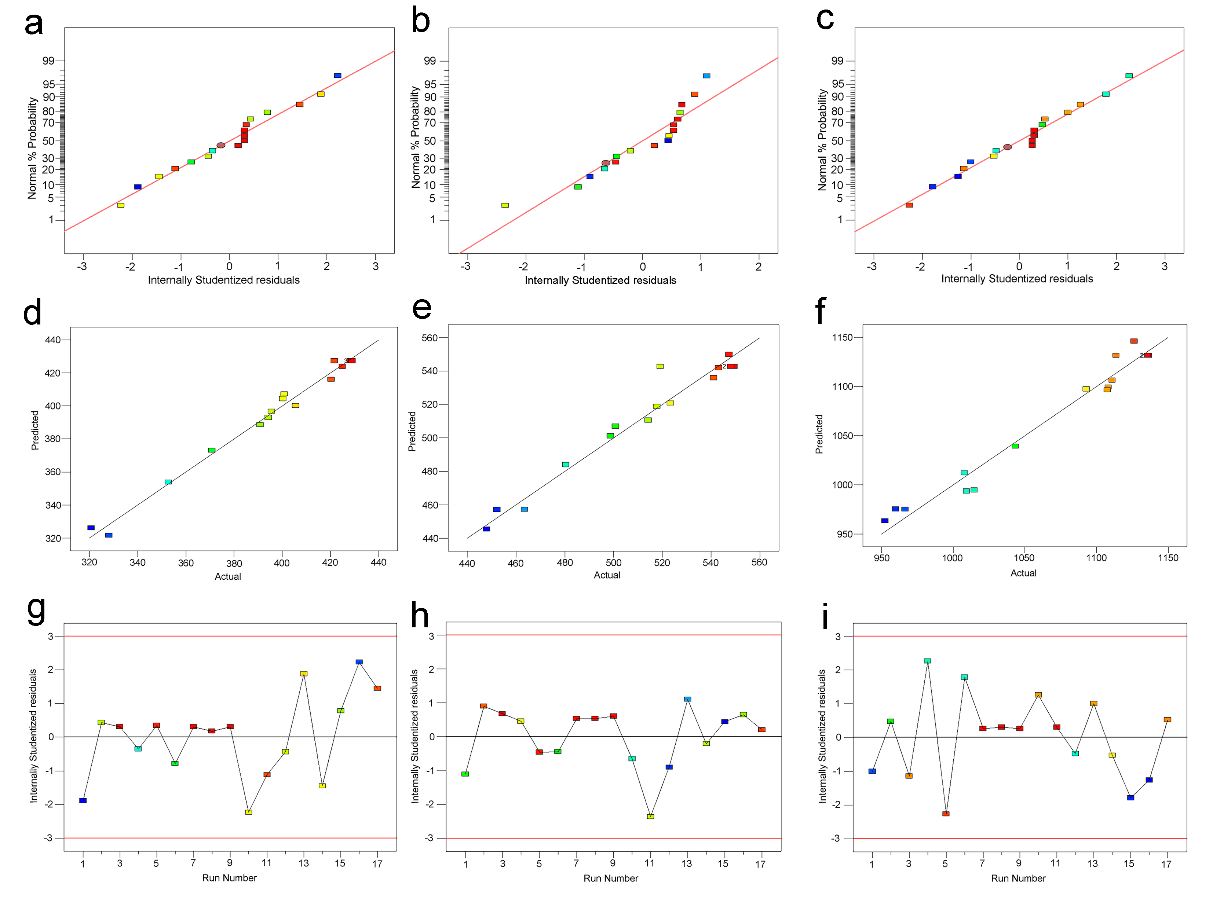


Figure S3 Three diagnostic plots for model adequacy survey for yield of quercetin (a, d, g) luteolin (b, e, h) and apigenin (c, f, i). Normal plot of residuals (a, b, c), the actual responses versus the predicted responses (d, e, f) and internally Studentized residuals versus run number (g, h, i).

3.14. Method Validation

The limits of detection (LODs) for quercetin, luteolin and apigenin were determined to be 0.0385, 0.0783, and 0.0748 mg/mL, respectively, while their corresponding limits of quantification (LOQs) were 0.1177, 0.2374, mg/mL and 0.2267 mg/mL, respectively. Stability and recovery studies demonstrated that the UACHEE extraction method coupled with HPLC analysis achieved optimal recovery rates for both analytes.

The stability and recovery studies of quercetin, luteolin and apigenin standards were evaluated under the following UACHEE conditions: ethanol volume fraction of 70%, 40 mg/g of dose of cellulase, 50 °C of incubation temperature, 148 min of incubation time, pH of 5, 20 mL/g of liquid‒solid ratio, duty cycle of 16.67%, 200 W of ultrasound irradiation power in the incubation process, 200 W of ultrasound irradiation power in the extraction process, and 15 min of ultrasound irradiation time in the extraction process. Table S1 showed that the average recovery of the standard solution of quercetin, luteolin and apigenin was 99.03%, 98.56% and 98.67%, the recovery of the recovered concentration of quercetin, luteolin and apigenin after 7 days were 97.22%, 97.56% and 96.84%, respectively. The results confirmed that no thermal isomerization or degradation occurred under the predicted operational parameters.

Method accuracy was assessed by spiking male inflorescences of *P. alba × berolinensis* samples with quercetin, luteolin and apigenin standard solutions at low, medium and high concentrations, followed by HPLC analysis. The measured quercetin, luteolin and apigenin contents were used to calculate recovery rates, which were respectively 98.11%, 98.14% and 98.42%, confirming the accuracy of the method. Method precision was confirmed by intraday and interday assays (RSD < 2%). Detailed precision data are provided in Table S1, which supported the reproducibility of the method.

Table S1 Method validation studies

| Stability studies of quercetin, luteolin and apigenin standards under the following UACHEE^a^ conditions: ethanol volume fraction of 70%, 40 mg/g of dose of cellulase, 50 °C of incubation temperature, 148 min of incubation time, pH of 5, 20 mL/g of liquid‒solid ratio, duty cycle of 16.67%, 200 W of ultrasound irradiation power in the incubation process, 200 W of ultrasound irradiation power in the extraction process, and 15 min of ultrasound irradiation time in the extraction process. | | | | | | | | | | | | | | | | | | | | | | | | | | |
| --- | --- | --- | --- | --- | --- | --- | --- | --- | --- | --- | --- | --- | --- | --- | --- | --- | --- | --- | --- | --- | --- | --- | --- | --- | --- | --- |
| Compounds | | Initial  concentration  (mg/mL) | | | Recovered  concentration  after UACHEE  (mg/mL) | | | | RSD% (n = 3) | | | Average  recovery (%) | | | | Recovered  concentration  after 7 days  (mg/mL) | | | RSD%  (n = 3) | | | | Average  recovery (%) | | | |
| Quercetin | | 0.1085 | | | 0.1079 | | | | 0.98 | | | 99.03 | | | | 0.1058 | | | 0.99 | | | | 97.22 | | | |
| Luteolin | | 0.1090 | | | 0.1085 | | | | 0.97 | | | 98.56 | | | | 0.1061 | | | 0.97 | | | | 97.56 | | | |
| Apigenin | | 0.1098 | | | 0.1094 | | | | 0.98 | | | 98.67 | | | | 0.1077 | | | 0.98 | | | | 96.84 | | | |
| Recovery of quercetin, luteolin and apigenin from the male inflorescences of *Populus alba* × *berolinensis* | | | | | | | | | | | | | | | | | | | | | | | | | | |
| Sample | Content of the  sample (mg) | | | | | | | Mass of added  standard (mg) | | | | | Mass of the sample analyzed with added standard (mg) | | | | | | | | Recovery (%) | | | | | |
|  | Quercetin | | Luteolin | | | Apigenin | | Quercetin | Luteolin | Apigenin | | | Quercetin | | Luteolin | | | Apigenin | | | Quercetin | | | Luteolin | | Apigenin |
| 1 | 1.16 | | 1.32 | | | 2.15 | | 0.50 | 0.50 | 2.00 | | | 1.60 | | 1.78 | | | 4.22 | | | 96.39 | | | 97.80 | | 97.68 |
| 2 | 1.16 | | 1.32 | | | 2.15 | | 1.00 | 1.00 | 3.00 | | | 2.14 | | 2.30 | | | 5.06 | | | 99.07 | | | 99.13 | | 98.25 |
| 3 | 1.16 | | 1.32 | | | 2.15 | | 1.50 | 1.50 | 4.00 | | | 2.63 | | 2.75 | | | 6.11 | | | 98.87 | | | 97.51 | | 99.34 |
| Average | | | | | | | | | | | | | | | | | | | | | 98.11 | | | 98.14 | | 98.42 |
| Precision results as peak area of different determinations on 3 different days, (quercetin, luteolin and apigenin Quercetin 0.1085 mg/mL, Luteolin 0.1090 mg/mL, Apigenin 0.1098 mg/mL (n = 3), acceptance limit RSD% < 2) | | | | | | | | | | | | | | | | | | | | | | | | | | |
| Sample | | | | Day 1 | | | | | | | Day 2 | | | | | | | | | Day 3 | | | | | | |
|  |  |  |  | Quercetin | | | Luteolin | | Apigenin | | Quercetin | | | Luteolin | | | Apigenin | | | Quercetin | | Luteolin | | | Apigenin | |
| 1 | | | | 4297 | | | 5215 | | 11335 | | 4268 | | | 5186 | | | 11305 | | | 4211 | | 5117 | | | 11267 | |
| 2 | | | | 4290 | | | 5106 | | 11300 | | 4257 | | | 5067 | | | 11278 | | | 4213 | | 5025 | | | 11214 | |
| 3 | | | | 4185 | | | 5188 | | 11254 | | 4166 | | | 5158 | | | 11202 | | | 4106 | | 5131 | | | 11175 | |
| Mean | | | | 4257 | | | 5170 | | 11296 | | 4230 | | | 5137 | | | 11262 | | | 4177 | | 5091 | | | 11219 | |
| Standard deviation | | | | 62.74 | | | 56.77 | | 40.62 | | 56.99 | | | 62.21 | | | 53.41 | | | 61.21 | | 57.58 | | | 46.18 | |
| RSD% | | | | 1.47 | | | 1.10 | | 0.36 | | 1.32 | | | 1.21 | | | 0.47 | | | 1.47 | | 1.13 | | | 0.41 | |

^a^ UACHEE: Ultrasound-assisted cellulase hydrolysis and ethanol extraction

3.15.4. Possible mechanism of extraction of quercetin, luteolin and apigenin by UACHEE

Compared with other pressing methods, the prominent advantage of the use of UACHEE to extract target components is the dual role of the cavitation phenomena of ultrasonic treatment and cellulase hydrolysis.

Cavitation is a mechanical effect generated by pressure changes in a liquid medium under the action of ultrasonic irradiation, and the cavitation phenomenon is subjected to different stages, including the formation, growth, collapse or implosion of bubbles^[55]^. When the ultrasound system was working, the sinusoidal ultrasonic waves were converted to mechanical vibrations, which led to compression and rarefaction in the liquid medium. The local pressure changes produce tiny gas bubbles. When the bubbles grow to a critical size, they implode and thus form a cavitation phenomenon, which is the most important effect of high-power ultrasound^[56]^. Moreover, the ultrasonic waves are transformed into thermal energy, resulting in a thermal effect, which accelerates the dissolution of the inner components of the target into the extraction solvent. Several mechanisms, including erosion, capillarity or sonoporation, have been shown to affect the process of ultrasonic-assisted extraction, thereby facilitating the breakdown of plant cell walls and the subsequent release and solubilization of target compounds into the solvent^[35]^.

On the other hand, cellulose is the main component of plant cell walls, and cellulase hydrolysis can fracture the *β*-glucoside bonds of cellulose, which could be effective in modifying the structure of plant cell walls and the dissolution of flavonoids^[57]^. The SEM, FTIR and XRD results also clarified the possible extraction mechanism of UACHEE to some extent. Furthermore, compared with conventional extraction techniques, ultrasound-enhanced enzymatic hydrolysis has shown superior extraction efficiency, achieving increased yields at reduced temperatures and shorter processing times.


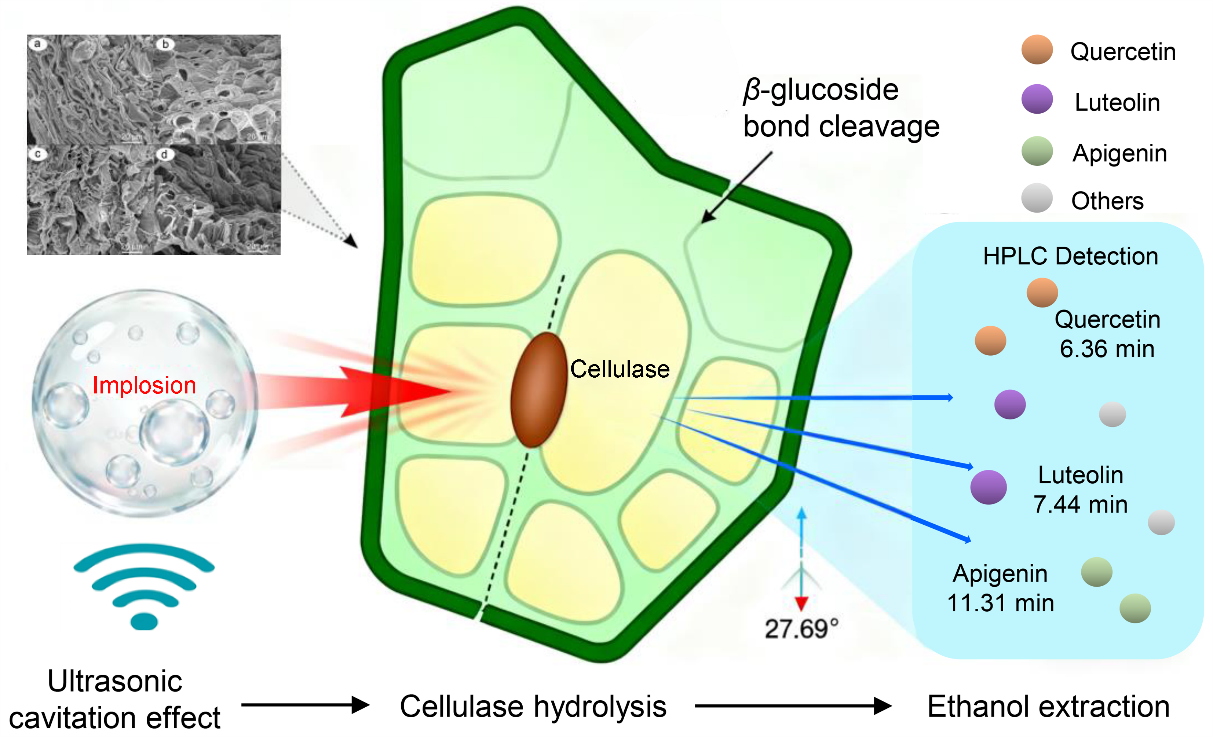


Figure S4 The mechanism diagram of UACHEE
